# Selective thoracolumbar/lumbar fusion for Syringomyelia-associated scoliosis: a case-control study with Lenke 5C adolescent idiopathic scoliosis

**DOI:** 10.1186/s12891-020-03779-0

**Published:** 2020-11-14

**Authors:** Fan Feng, Hongxing Shen, Xiuyuan Chen, Zude Liu, Jianwei Chen, Quan Li, Lifeng Lao

**Affiliations:** Department of Spine Surgery, Renji Hospital, Shanghai Jiaotong University School of Medicine, No.160 Pujian Road, Shanghai, 200120 China

**Keywords:** Syringomyelia, Scoliosis, Selective fusion, Thoracolumbar/lumbar

## Abstract

**Background:**

Selective thoracolumbar/lumbar fusion technique was introduced to treat adolescent idiopathic scoliosis (AIS) patients with major thoracolumbar/lumbar curves. Theoretically, this surgical strategy could also be applied to syringomyelia patients. No previous study has specifically addressed the effectiveness of selective thoracolumbar/lumbar fusion for patients with syringomyelia-associated scoliosis. The aim of the study was to investigate the effectiveness of selective thoracolumbar/lumbar fusion for the surgical treatment of patients with syringomyelia-associated scoliosis.

**Methods:**

From February 2010 to September 2016, 14 syringomyelia-associated patients with major thoracolumbar/lumbar curves were retrospectively reviewed. Besides, 30 Lenke 5C AIS patients were enrolled as a control group. Posterior selective thoracolumbar/lumbar fusion was performed for both groups. Patients’ demographic, operative, radiological, and quality of life data were reviewed with follow-up. Intragroup comparisons were performed for each parameter.

**Results:**

The two groups were matched by age, gender, curve characteristics, duration of follow-up, and all preoperative radiographic parameters except for thoracic kyphosis. After surgery, the average correction rate of the major thoracolumbar/lumbar curve was 82.2 ± 7.8% in the syringomyelia group, which was not significantly different from that of AIS group (82.5 ± 10.6%, *P* = 0.47). A similar improvement of unfused thoracic curve was observed between the two groups (50.1 ± 16.5% vs. 48.5 ± 26.9%, *P* = 0.29). During the follow-up, the correction effect of scoliosis was well maintained, without aggravation of the original neural symptoms or fresh permanent neurological deficits. Of note, the number of fusion levels was significantly larger in syringomyelia group than that in AIS group (7.6 ± 1.4 vs. 6.5 ± 1.2, *P* < 0.01). The average follow up was 47.6 months (36–81 months).

**Conclusion:**

Similar to AIS cases, syringomyelia-associated scoliosis can be effectively and safely corrected by selective thoracolumbar/lumbar fusion with satisfactory surgical outcomes. However, the syringomyelia group, on average, required an additional fused segment for treatment as compared to the AIS group (7.6 versus 6.5 in the AIS group).

**Supplementary Information:**

The online version contains supplementary material available at 10.1186/s12891-020-03779-0.

## Background

According to the classification system provided by the Scoliosis Research Society (SRS), scoliosis has etiologically been classified as idiopathic, congenital, neuromuscular, and syndromic [1, 2]. Syringomyelia-associated scoliosis is a common type of neuromuscular scoliosis [3]. Syringomyelia is an aetiologically diverse affliction characterized by a longitudinally oriented fluid-containing cavity that anatomically lies within the spinal cord parenchyma and is often associated with spinal deformity [4, 5]. Syringomyelia-related scoliosis is often called neurogenic, based on the hypothesis that spinal cord dysfunction due to central canal dilation may cause a spinal deformity. Zhu et al. [6] revealed a significant correlation between curve convexity and the dominant side of a deviated syrinx, with an 83.3% concordance rate. Godzik and co-workers [7] noted that Chiari-I malformation without syringomyelia was associated with scoliosis and that these patients had fewer atypical curve features, including fewer left curves and fewer kyphotic curves, than patients who had a Chiari-I malformation with syringomyelia. The actual aetiological mechanisms of syringomyelia remain obscure. Many previous studies have reported an “idiopathic” curve in syringomyelia patient, thus it seems reasonable that patients with syringomyelia-associated scoliosis could theoretically be treated with the same guideline applied for adolescent idiopathic scoliosis (AIS).

Achieving global spinal balance, preventing curve progression, and saving mobile segments are major goals of corrective surgery in AIS cases. Selective fusion has gained popularity over decades [8–10]. In recent studies, posterior selective thoracic fusion has been proven to produce a satisfying surgical outcome in syringomyelia patients with major thoracic curve [11–13]. However, to the best of our knowledge, no study has specifically focused on the thoracolumbar/lumbar curve in patients with syringomyelia-associated scoliosis. According to a recent study, syringomyelia patients with major thoracic and major thoracolumbar/lumbar curves may have different syrinx features [14]. Due to these disparities, it is still not clear whether syringomyelia-associated thoracolumbar/lumbar scoliosis could be treated with the same guidelines applied for Lenke 5C AIS. To address these issues, we compared the radiographic outcomes of posterior spinal fusion between syringomyelia patients and age, sex, and curve-magnitude matched AIS patients in this retrospective series. The objective of this study is to investigate the effectiveness of selective thoracolumbar/lumbar fusion in patients with syringomyelia-associated scoliosis.

## Methods

After obtaining the approval from the institutional review board of our hospital, two groups of scoliosis patients who underwent posterior spinal fusion surgeries from February 2010 to September 2016 were retrospectively reviewed. Syringomyelia group was composed of 14 patients with syringomyelia-associated scoliosis. The inclusion criteria were as follows: (1) patients aged from 12 to 18 years at surgery, (2) a major thoracolumbar/lumbar curve with a nonstructural thoracic curve comparable to Lenke 5C AIS [9], (3) patients who underwent posterior selective thoracolumbar/lumbar fusion; and (4) a minimum of 3-years of follow-up. Patients with spinal revision surgery, congenital vertebra deformities or incomplete follow-up information were excluded from the study. Specifically, patients with syringomyelia-associated scoliosis were first evaluated by a neurological surgeon to decide whether surgical procedures were needed to treat neurological abnormalities and reduce the risk of postoperative neurological complications. For patients undergoing neurosurgical treatment of their syrinx, correction surgeries for scoliosis were performed 6 months later.

A total of 30 age, sex, and curve-magnitude matched patients with Lenke 5C AIS were assigned to AIS group as controls. Their main thoracic and upper thoracic curves were nonstructural, meaning that their magnitude was less than that of the structural thoracolumbar/lumbar curve and less than 25° on convex side-bending radiographs, and sagittal kyphosis was absent (T10-L2 and T2-T5 were less than 20°). All patients in this study underwent selective thoracolumbar/lumbar fusion with a minimum of 2 years of follow-up.

### Radiographic measurements

Standing posteroanterior and lateral radiographs obtained preoperatively, postoperatively, and at the last follow-up were evaluated by an independent observer. Radiographic parameters include thoracic and lumbar Cobb angles, apical vertebral translation (AVT), trunk shift, thoracic kyphosis (TK), lumbar lordosis (LL), and sagittal vertical axis (SVA). AVT was defined as the distance from the midpoint of the lumbar apical vertebra to the center sacral vertical line. Trunk shift was determined by the perpendicular distance between the midpoint of S1 and a plumb line drawn from C7 on standing posteroanterior radiographs. A patient was considered to have coronal decompensation if they had a trunk shift ≥20 mm. TK was evaluated by the angle between the upperendplate of T5 and the lower endplate of T12. LL was assessed by the Cobb angle between the two lines through the superior endplate of L1 and S1. The angle was positive when the curve was kyphotic and negative when the curve was lordotic. The SVA was defined as the distance between the C7 plumb line and the posterior superior corner of S1, which has a positive value when the vertical plumbline lies anterior to the posterior superior corner of S1. Using the lateral bending radiographs taken before surgery, the curve flexibility was calculated according to the following formula: percentage of flexibility = (preoperative Cobb angle - bending Cobb angle)/preoperative Cobb angle *100%. The type of syrinx was classified into the four types, distended, moniliform, slender and circumscribed, based on the sagittal MRI findings [5]. Chiari-I malformation was defined as tonsillar herniation of at least 5 mm below the foramen magnum on the MR images [15].

### Evaluation of health-related quality of life

Patients with syringomyelia-associated scoliosis were all required to complete the SRS-22 questionnaires before surgery and at the final follow-up, respectively. The SRS-22 covers five domains including function, pain, self-perceived image, satisfaction with treatment, and mental health. Questions of each domain have five verbal response alternatives ranging from 1 to 5, with a value of 5 indicating the best outcome.

### Statistical analysis

The SPSS 21.0 software was used for the statistical analysis. Differences between the groups were compared using independent sample t-tests and chi-square tests. Paired-sample t tests were used to compare the changes between preoperative and postoperative radiographic measurements. Besides, the comparison of the SRS-22 questionnaire scores between preoperatively and the last follow-up were performed with the Student t test. The significance level was set at *P* < 0.05.

## Results

### Demographic characteristics

**Fig. 1:**
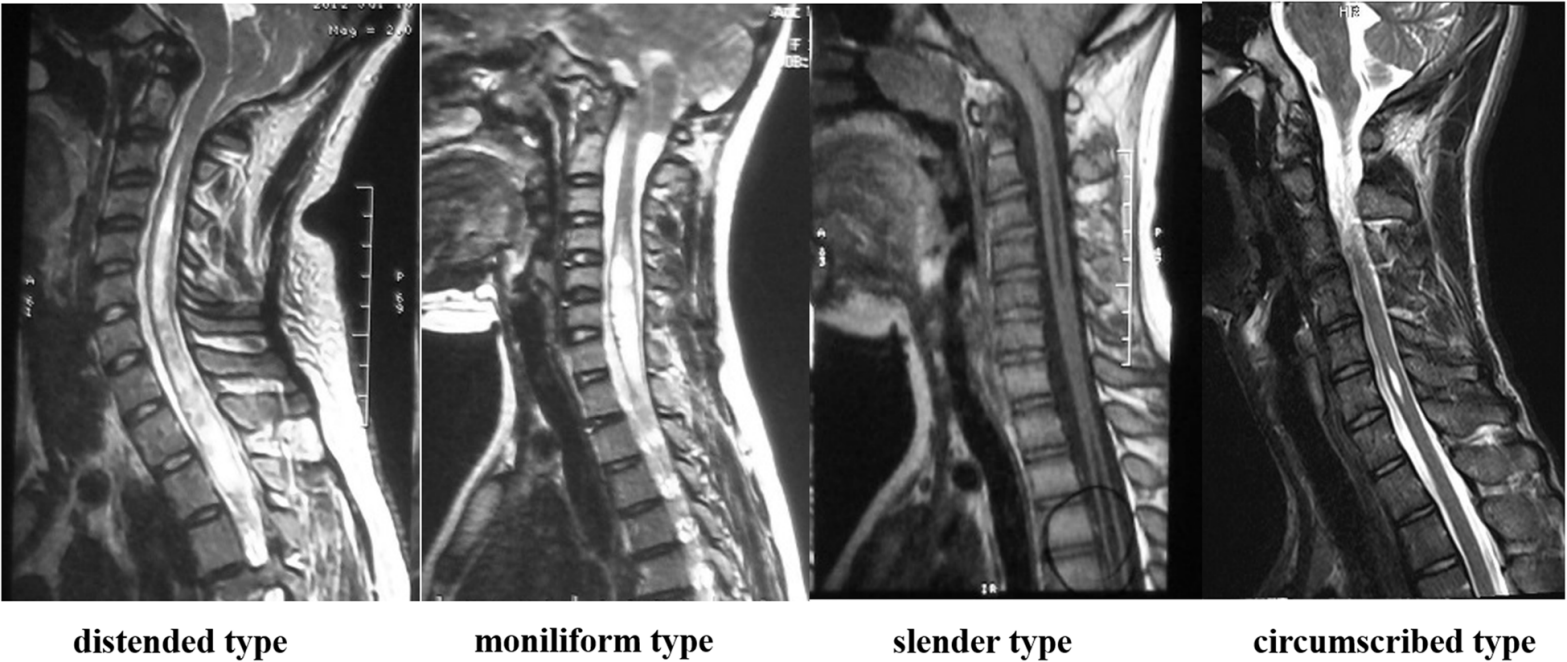
The classification of the syrinx

Syringomyelia group consisted of 14 patients (6 males and 8 females) diagnosed with syringomyelia-associated scoliosis with thoracolumbar/lumbar (5 left and 9 right) curves. Among these patients, 3 (21.4%) syrinxes were located in the cervical spine, 8 (57.1%) crossed the cervicothoracic junction, and 3 (21.4%) were located in the thoracic region. Regarding the classification of the syrinx [5] (Fig. 1), a distended type syrinx was found in 2 patients (14.3%), a moniliform type syrinx was found in 1 patient (7.1%), a slender type syrinx was found in 8 patients (57.1%) and a circumscribed type was found in 3 patient (21.4%). Five patients were found to have coexisting Chiari I malformation. With regard to preoperative neurological status, 8 patients reported abnormal reflex (diminished abdominal reflexes and abnormal tendon reflex), 2 patients had positive Babinski sign, and 2 patients complained of headache or back pain. Prior to the corrective surgery for scoliosis, 3 patients underwent posterior fossa decompression surgery and 3 patients received a syrinx shunt. All patients experienced resolution of their neurological deficits 6 months after neurosurgical intervention. All clinical details for these 14 patients are shown in Table 1.

**Table 1:** Preoperative characteristics in syringomyelia-associated patients with major thoracolumbar/lumbar curves

Patient No.	Gender/Age	Level of syringomyelia	Type of syringomyelia	Preoperative neurological status	Major curve (°)	Fusion level	Follow-up (mo)
1	Female/17	C4–7	slender	diminished abdominal reflexes and abnormal tendon reflex	42.7	T11-L4	81
2	Male/18	C2-T2	slender	headache and abnormal tendon reflex	37.4	T9-L4	36
3	Female/16	C1-T5	moniliform	back pain, abnormal tendon reflex and positive Babinski sign	55.3	T8-L4	48
4	Male/11	C1-T10	slender	negative	41.2	T8-L3	64
5	Female/15	T5-T8	slender	negative	40.5	T11-L4	37
6	Female/11	C3-T4	distended	sensor deficit and abnormal tendon reflex	41.6	T10-L3	48
7	Male/17	C4-T9	slender	abnormal tendon reflex and positive Babinski sign	42.4	T9-L3	54
8	Female/12	C4-T1	distended	diminished abdominal reflexes	44.9	T7-L3	36
9	Female/13	C4-C7,T5-T8	slender	negative	52.8	T7-L3	48
10	Male/11	T1-T12	slender	diminished abdominal reflexes, abnormal tendon reflex	76.7	T7-L4	39
11	Male/16	C5–6	circumscribed	negative	45.7	T10-L4	36
12	Female/14	T3-T7	slender	diminished abdominal reflexes	46.3	T10-L4	47
13	Female/15	C5-C7,T2-T4	circumscribed	negative	43.5	T11-L4	52
14	Male/13	C4–7	circumscribed	negative	42.6	T8-L3	40

### Preoperative measurements

Preoperative radiographic measurements of the two groups are summarized in Table 2. The two groups were matched in terms of age, sex, Cobb angle of the major curve, surgical approach and duration of follow-up. For patients with syringomyelia-associated scoliosis, the mean preoperative Cobb angle of the thoracolumbar/lumbar curve was 46.7 ± 9.8° with a mean flexibility of 58.6 ± 20.6%. The mean preoperative Cobb angle of the thoracic curve was 23.9 ± 10.4° with a mean flexibility of 73.2 ± 29.2%. As shown in Table 2, all preoperative radiographic measurements except for TK were comparable between the two groups.

**Table 2:** Preoperative radiographic measurements in two groups

parameters	Syringomyelia group	AIS group	***P*** value
	(*n* = 14)	(*n* = 30)	
Age (yrs)	14.2 ± 2.4	15.0 ± 1.8	0.25
Sex (M/F)	6/8	6/24	0.11
TL/L-Cobb (°)	46.7 ± 9.8	47.0 ± 9.5	0.73
TL/L-flexibility (%)	68.6 ± 20.5	70.9 ± 18.7	0.30
TL/L -AVR (°)	2.0 ± 0.8	2.0 ± 0.6	0.84
TL/L -AVT (mm)	47.8 ± 12.3	46.4 ± 14.2	0.50
T-Cobb (°)	23.9 ± 10.4	25.1 ± 6.4	0.27
T-flexibility (%)	73.2 ± 29.2	72.7 ± 23.8	0.61
T-AVR (°)	0.9 ± 0.5	0.8 ± 0.4	0.82
T-AVT (mm)	11.2 ± 7.0	12.6 ± 6.2	0.52
TS (mm)	17.9 ± 11.0	19.4 ± 11.3	0.29
TK (T5-T12, °)	23.6 ± 9.9	19.9 ± 8.7	0.03*
LL (L1-S1, °)	50.9 ± 10.0	50.2 ± 14.1	0.46
SVA (mm)	25.9 ± 27.0	27.9 ± 23.5	0.34

*AVR* Apical vertebral rotation, *AVT* Apical vertebral translation, *TS* Trunk shift, *TK* Thoracic kyphosis, *LL* Lumbar lordosis, *SVA* Sagittal vertical axis

* means statistically significant difference between two groups (*P* < 0.05)

### Correction outcomes

**Fig. 2:**
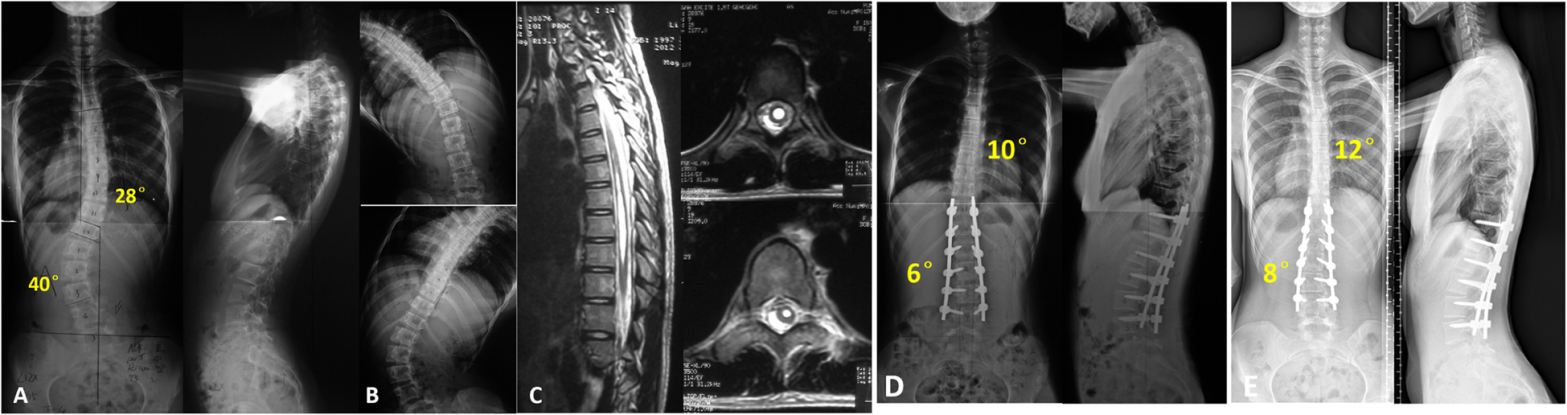
15-year-old girl with a main lumbar curve secondary to syringomyelia. The radiographs demonstrate a lumbar curve of 40° and a thoracic curve of 28° (**a**). Preoperative bending radiograph shows the high flexibility of major lumbar curve and minor thoracic curve (**b**). Sagittal and axial views of the syrinx (T5-T8) (**c**). Postoperative posteroanterior and lateral radiographs following posterior selective lumbar fusion from T11 to L4 demonstrate satisfying correction of syringomyelia-associated scoliosis (**d**). Radiographs made at three years follow-up (**e**) show that correction outcome had been well maintained

**Fig. 3:**
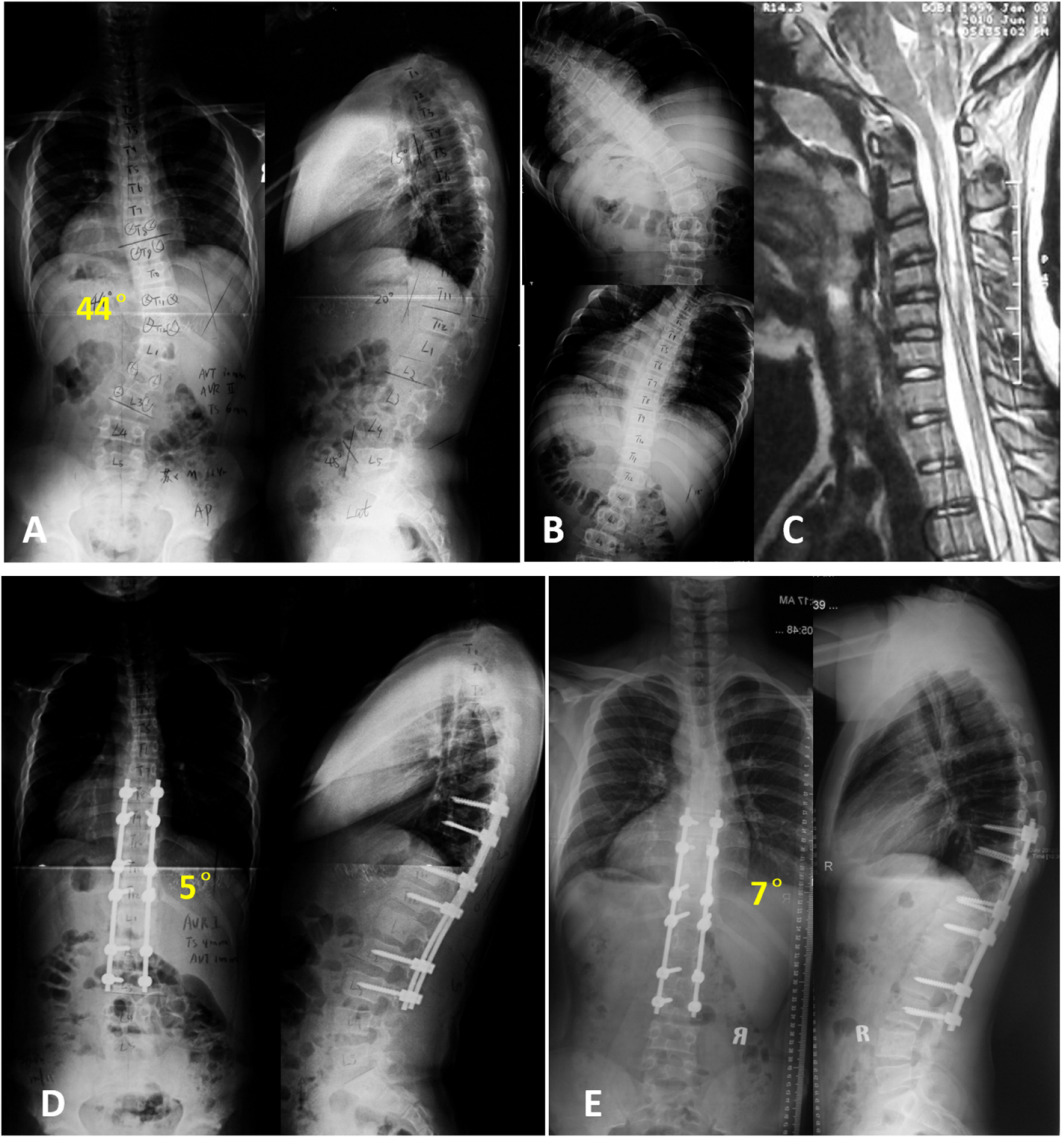
12-year-old boy with a major thoracolumbar curve (44°) secondary to syringomyelia (**a**). Preoperative bending radiograph shows the high flexibility of major thoracolumbar curve (**b**). Sagittal views of the syrinx located at cervical and thoracic region (slender type) (**c**). Posterior selective thoracolumbar fusion from T8 to L3 achieved effective correction of scoliosis (**d**). Radiographs made at five years follow-up (**e**)

The average follow up was 47.6 months (36–81 months). The coronal Cobb angle of the thoracolumbar/lumbar curve was corrected to 8.3 ± 5.3° postoperatively with an immediate correction rate of 82.2 ± 7.8%, with a correction loss of 4.4 ± 3.7° at final follow-up. The non-structural thoracic curve was corrected to 11.9 ± 5.8° immediately postoperatively with a spontaneous correction of 50.1 ± 16.5%, with a correction loss of 3.5 ± 6.3° at final follow-up. Moreover, the AVT and AVR of both the TL/thoracolumbar/lumbar curve and thoracic curve were significantly decreased after surgery. Trunk shift was also improved to 18.6 ± 20.4 mm postoperatively and 6.9 ± 7.4 mm at final follow-up. In the sagittal plane, both thoracic kyphosis, lumbar lordosis and SVA were maintained and tended to be consistent with the physiological curve. During the follow-up period, the scoliosis correction maintained well without sign of internal fixation failure or pseudarthrosis needed for revision surgery. (Figs. 2 and 3).

The results of intergroup comparisons of surgical outcomes between the two groups are summarized in Table 3. Postoperative radiographic measurements revealed a comparable correction rate of the major thoracolumbar/lumbar curve in both groups (82.2 ± 7.8% vs. 82.5 ± 10.6%, *P* = 0.47). A similar improvement in the thoracic curve was also observed between the two groups (50.1 ± 16.5% vs. 48.5 ± 26.9%, *P* = 0.29). With regard to other radiographic parameters, including the AVT of both the thoracolumbar/lumbar curve and thoracic curve, trunk shift, TK, LL and sagittal balance as indicated by SVA, no significant differences were observed between the two groups (*P* > 0.05).

**Table 3:** Comparison of surgical outcomes between two groups

	Syringomyelia group	AIS group	*P*
No. of levels fused	7.6 ± 1.4	6.5 ± 1.2	< 0.01*
LIV (L3/L4)	6/8	13/17	0.98
Postoperative TL/L –Cobb (°)	8.3 ± 5.3	6.9 ± 4.2	0.09
Postoperative TL/L-AVT (mm)	13.5 ± 7.7	12.9 ± 8.4	0.52
TL/L-correction rate (%)	82.2 ± 7.8	82.5 ± 10.6	0.47
TL/L -correction loss (°)	4.4 ± 3.7	3.9 ± 4.6	0.76
Postoperative T-Cobb (°)	11.9 ± 5.8	13.2 ± 6.7	0.21
Postoperative T-AVT (mm)	13.7 ± 7.2	15.4 ± 8.6	0.51
T-correction rate (%)	50.1 ± 16.5	48.5 ± 26.9	0.29
T-correction loss (°)	3.4 ± 6.3	2.7 ± 4.3	0.43
Postoperative TS (mm)	18.6 ± 20.4	18.3 ± 15.1	0.85
Postoperative TK (°)	24.1 ± 9.7	23.6 ± 8.5	0.44
Postoperative LL (°)	54.9 ± 10.8	53.6 ± 12.1	0.63
Postoperative SVA (mm)	18.4 ± 17.3	20.2 ± 16.8	0.23
Follow-up period (month)	47.6 ± 12.7	46.3 ± 13.8	0.42

* means statistically significant difference between two groups (*P* < 0.05)

However, the number of fusion levels was significantly larger in syringomyelia group compared to AIS group (7.6 ± 1.4 vs. 6.5 ± 1.2, *P* < 0.01). All lowest instrumented vertebrae (LIV) were located at L3 or L4, and no significant difference in LIV distribution was found between the two groups (*P* = 0.98). However, there were statistical difference for upper instrumented vertebrae (UIV) between two groups (χ^2^ = 10.222, *P* = 0.037). In AIS group, UIV was T8 (2 cases), T9 (4 cases), T10 (13 cases) and T11 (11 cases). Six cases (20%) underwent short segment fusions (5 levels). Whereas in the syringomyelia group, all cases had at least 6 fusion levels, UIV was T7 (3 cases), T8 (3 cases), T9 (2 cases), T10 (3 cases) and T11 (3 cases).

### Health-related quality of life results

In syringomyelia group, all five domains of the SRS-22 questionnaire showed a general improvement overall. Statistically, the total SRS-22 score (*P* = 0.03), self-image domain score (*P* < 0.01) and the satisfaction domain score (*P* = 0.02) demonstrated significant difference compared with preoperative data (Table 4).

**Table 4:** Score of SRS-22 for syringomyelia-associated scoliosis patient with major thoracolumbar/lumbar curve

	Preoperatively	Final follow-up	*P*
Pain domain	3.8 ± 0.5	4.2 ± 0.4	0.07
Function domain	3.9 ± 0.6	4.1 ± 0.5	0.26
Self-image domain	3.6 ± 0.5	4.3 ± 0.6	< 0.01*
Mental health domain	4.0 ± 0.4	4.2 ± 0.3	0.58
Satisfaction domain	3.8 ± 0.7	4.3 ± 0.4	0.02*
total	3.8 ± 0.4	4.2 ± 0.3	0.03*

* means statistically significant difference calculated by Student t test

### Complications

In syringomyelia group, one patient had transient SEP signal change and mild postoperative numbness in left lower extremity. Postoperative CT and MRI examinations did not reveal any malposition of pedicle screws or hematoma. He was conservatively treated with mecobalamin and glucocorticoids, and the numbness symptom completely disappeared after 4 days. During the follow-up period, none of the patients showed aggravation of the original neural symptoms or fresh permanent neurological complication in both groups. More details of cases were shown in Supplementary Information (Additional file 1).

## Discussion

As a common type of neuromuscular scoliosis, the rate of scoliosis in syringomyelia patients ranges from 25 to 74.4% [16]. Rodriguez et al. [17] found that 49.1% of cases were “idiopathic” scoliosis among IS patients. Many studies have reported that syringomyelia associated scoliosis was unintentionally found in “presumed” idiopathic scoliosis [18, 19]. To date, relatively few peer-reviewed articles have published the clinical outcomes of surgical correction for patients with syringomyelia-associated scoliosis. Bradley et al. [20] reported an average correction rate of 48% (6–83%) in 13 syringomyelia patients whose spinal deformities were safely corrected according to idiopathic criteria. Zhang et al. [21] demonstrated 13 cases of thoracolumbar or lumbar syringomyelia-related scoliosis with an average correction rate of 80.7%. Sha and co-workers [22] carefully reviewed 69 syringomyelia-associated thoracic scoliosis cases and compared the outcomes of spinal instrumentation and fusion between those with syringomyelia-related deformity and those with AIS. Qin et al. [12] demonstrated that syringomyelia-associated scoliosis can be successfully corrected through selective thoracic fusion surgery with a promising long-term surgical outcome. Therefore, patients with syringomyelia-associated thoracic scoliosis can be treated with similar surgical strategies to those applied to idiopathic scoliosis when an idiopathic-like curvature was present [23]. Although single thoracic scoliosis is the predominant curve type in syringomyelia-associated scoliosis [24], some patients present with major thoracolumbar/lumbar curve. Recently, Tan and co-workers [14] showed a remarkable observation that syringomyelia patients with major curves located in the thoracolumbar or lumbar spine had unique features of their syrinx, in which their syrinx locations were much lower caudally and produced greater dilation of the spinal cord (syrinx/cord ratio) than in patients with major curves located in the thoracic spine. Previous studies revealed a significant correlation between curve convexity and the dominant side of a deviated syrinx [6], which would be supportive of the hypothesis that patients with a major thoracolumbar/lumbar curve might have different scoliosis characteristics. According to this hypothesis, a crucial question is whether the surgical strategy used for Lenke5C AIS can be applied to syringomyelia patients with a major thoracolumbar/lumbar curve. There is still a lack of consensus on whether patients with syringomyelia-associated scoliosis can be corrected with selective thoracolumbar or lumbar fusion. A well-designed case-control study is therefore warranted to address this issue. In present study, the effectiveness of selective thoracolumbar/lumbar fusion in patients with syringomyelia-associated scoliosis was investigated through comparison with another group of patients with AIS undergoing the same surgical strategy. To our knowledge, this is the first clinical study to investigate the feasibility of applying selective thoracolumbar/lumbar fusion for the correction of idiopathic-like scoliosis secondary to syringomyelia.

In the current study, the correction rate of the thoracolumbar/lumbar curve and compensatory thoracic curve in syringomyelia patients was found to be comparable to that of Lenke 5C AIS patients. The postoperative correction rate of patients with syringomyelia-associated scoliosis was 82.2% on average for major thoracolumbar/lumbar curves. It slightly exceed the curve flexibility (68.6%), preventing excessive traction to the spinal cord and yielding a good corrective outcomes. Owing to the favorable preoperative Cobb angle (less than 30°) and high flexibility (more than 70%), the minor thoracic curve was also spontaneously corrected with an average rate of 50.1%. Moreover, the postoperative sagittal profile, including TK, LL and SVA were also comparable between the two groups. Regarding the low correction loss (4.4°), the correction effects of all 14 patients with syringomyelia associated scoliosis were well-maintained at the final follow-up, which demonstrated that syringomyelia-associated scoliosis can be effectively corrected by selective thoracolumbar/lumbar fusion.

Notably, the results of our study also showed an interesting observation that, in the syrinx-related cases, the fusion levels were longer than those of Lenke5C AIS patients (7.6 vs. 6.5). All syringomyelia patients had at least 6 fusion levels, which was in contrast with some AIS cases (6/30, 20%) with short segment fusions (5 levels). In both groups, we determine the fusion levels mainly based on the treatment guidelines of Lenke classification for AIS. As shown in Table 3, there was no statistical difference in the distribution of LIV in both groups (L3/L4, 6/8 in syringomyelia group and 13/17 in AIS group, *p* > 0.05). Generally, we chose the upper end vertebra (UEV) as UIV. However, there were slightly different surgical strategies for UIV in two groups. In AIS group, UIV was T8 (2 cases), T9 (4 cases), T10 (13 cases) and T11 (11 cases). In some AIS cases, to save as many mobile segments, UIV was selected as 1 level below the UEV. In AIS group, 6 cases (20%) underwent short segment fusions (5 levels). Whereas, short levels fusion may not be appropriate in patients with syringomyelia, considering the potential progression of the curve following fusion [20, 25]. In syringomyelia group, UIV was T7 (3 cases), T8 (3 cases), T9 (2 cases), T10 (3 cases) and T11 (3 cases). UIV was selected as 1 level above the UEV in six syringomyelia cases. For example, in the case of Fig. 2, UEV was T12 and UIV was T11. In the case of Fig. 3, UEV was T9 and UIV was T8. Therefore, the syringomyelia group had a larger number of fusion levels. Similarly, in Zhang et al. observation [21] 13 cases of thoracolumbar or lumbar scoliosis with Chiari malformation and syringomyelia underwent posterior pedicle screw instrumentation for the correction of scoliosis. Their average number of segments involved in fusion was eight (range, 7–10). Bradley et al. [20] proposed to fuse an increased number of vertebrae when planning scoliosis surgery secondary to syringomyelia. Our observation is consistent with the recommendation of Bradley et al. They suggested that short-segment fusion may be appropriate for AIS patients; however progression of the deformity in patients with syringomyelia following fusion requires a more extensive fusion. There is one possible explanation for this phenomenon: the mechanism underlying the development of scoliosis secondary to syringomyelia may also affect the surgical strategy. The imbalance of trunk muscles, which is caused by asymmetric pressure on grey matter from a deviated syrinx, can contribute to the development of scoliosis [20]. Local denervation of the axial musculature, in addition to loss of medial anterior horn cells from syringomyelia, could favour the development of spinal deformities. Therefore, the syringomyelia group may require an additional fused segment for treatment as compared to the AIS group.

In early studies, increased neurological complications from neuromuscular scoliosis surgery were reported in these patients [26, 27]. The vulnerable blood supply in the dilated cord, intraoperative traction force on already damaged neural tissue or changes in cerebrospinal fluid pressure after surgery have been suspected to increase the risk in these patients [28]. Godzik et al. [29] reviewed a matched cohort to compare surgery for idiopathic and syrinx-related curves and found that the syringomyelia group had more neurological complications (11% versus 0%). In recent decades, the evolution of instrumentation systems that allow correction without distraction has greatly improved the safety of surgical treatment for spinal deformities. Spinal cord monitors have played a significant role in preventing neurological complications during correction manoeuvres. Recent studies have demonstrated encouraging clinical outcomes showing that spinal corrective surgery can be safely performed for syringomyelia-associated scoliosis without producing a higher risk of neurological complications [12, 22, 30, 31]. In our practice, intraoperative spinal cord monitoring was performed for in each patient. It is still prudent to approach this surgery with an extra degree of caution regarding the amount of correction and to carefully interpret any abnormal changes on the spinal cord monitor. To reduce the neurological risk, we performed the correction procedure mainly by rod derotation technique and compression on the convex side, allowing minimal propping open at the concave side and minimal traction of the spinal nerves. In addition, vertebral derotation is relatively safe on the convex side, without infringement to the large vessels and internal organs. Although one patient experienced transient neural deficits, none of the patients suffered aggravation of the original neural symptoms or fresh permanent neurological complication in both groups.

The current study has few limitations. First, some authors believe in the syrinx-related cases, the dilated spinal cord receded following the spinal correction and fusion [22]. Whether straightening and immobilizing the spine alter spinal fluid dynamics remains unknown. Fortunately, in this series no fresh neurological deficits occurred or progressed during the follow-up. Further study with postoperative spinal MRI to evaluate the syrinx is warranted. Second, the number of thoracolumbar/lumbar scoliosis patients was relatively small due to the syringomyelia itself. However, our study has a major strength in that it included a very homogeneous group of patients. All patients were operated on by surgeons from the same institution and had similar operative indications and surgical techniques that were focused on selective thoracolumbar/lumbar fusion in syringomyelia-associated scoliosis.

## Conclusions

The present study, with the largest cohort to date, demonstrates that selective thoracolumbar/lumbar fusion can be effectively and safely performed in patients with syringomyelia-associated scoliosis. The radiological outcomes of surgery, including curve severity, balance, flexibility, overall correction, and loss of correction, revealed satisfactory and equivalent results in the syringomyelia group when compared to the AIS group. However, the syringomyelia group, on average, required an additional fused segment for treatment as compared to the AIS group (7.6 versus 6.5 in the AIS group).

## Supplementary Information

**Additional file 1:**

## Data Availability

The datasets used and/or analyzed during the current study are available from the corresponding author on reasonable request.

## Abbreviations

AIS: Adolescent idiopathic scoliosis
AVT: Apical vertebral translation
TK: Thoracic kyphosis
LL: Lumbar lordosis
SVA: Sagittal vertical axis
SRS-22: Scoliosis research society-22
LIV: Lowest instrumented vertebrae
SEP: Somatosensory evoked potentials
