# Supplementary material for: Selective thoracolumbar/lumbar fusion for Syringomyelia-associated scoliosis: a case-control study with Lenke 5C adolescent idiopathic scoliosis
Source: BMC Musculoskelet Disord. 2020 Nov 14;21:749. doi: 10.1186/s12891-020-03779-0 (PMC7666459; doi:10.1186/s12891-020-03779-0)
Supplement: Supplementary file 1 — Additional file 1: [file 12891_2020_3779_MOESM1_ESM.pptx]

## Slide 1
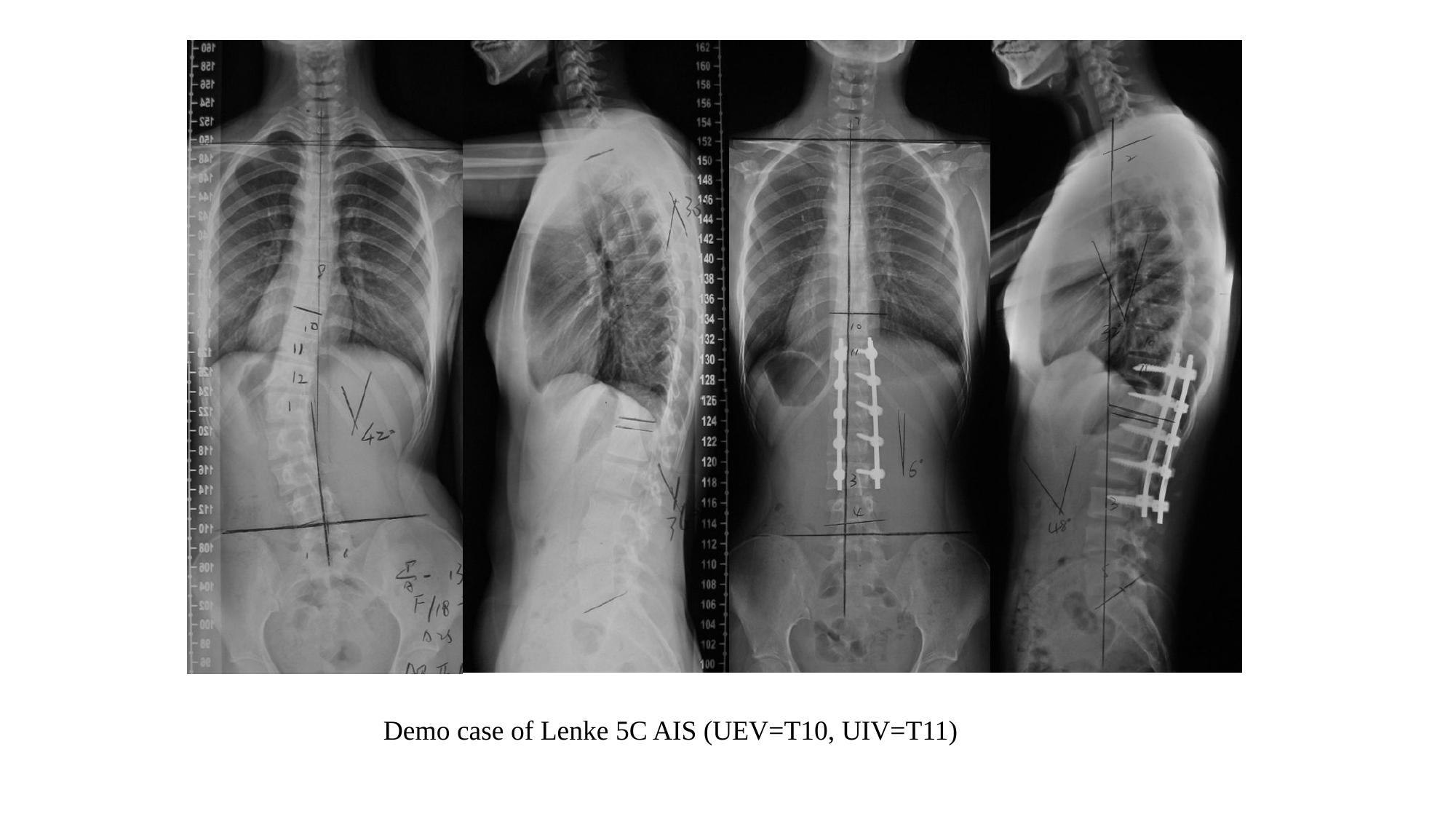

Demo case of Lenke 5C AIS (UEV=T10, UIV=T11)

## Slide 2
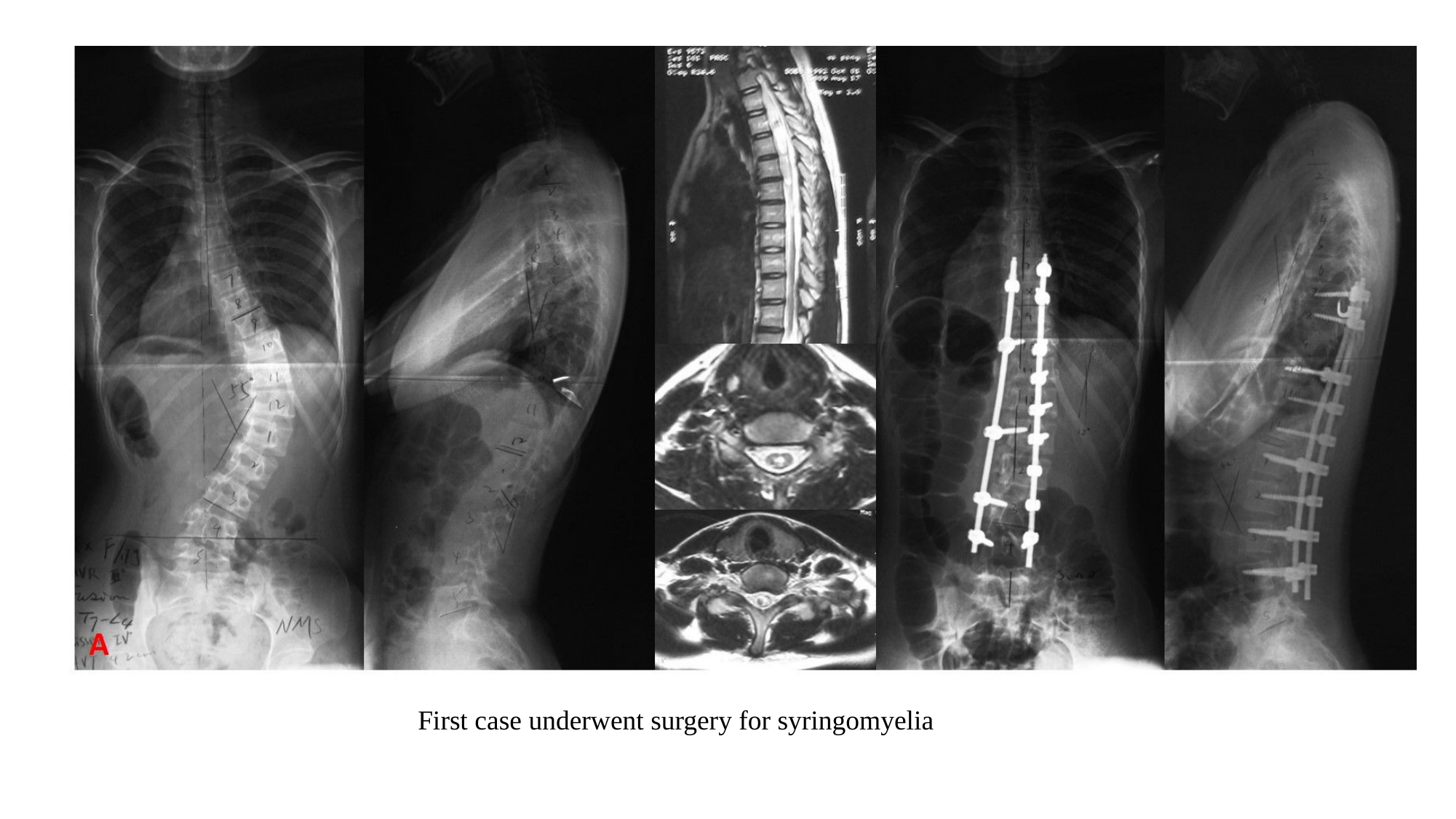

First case underwent surgery for syringomyelia

## Slide 3
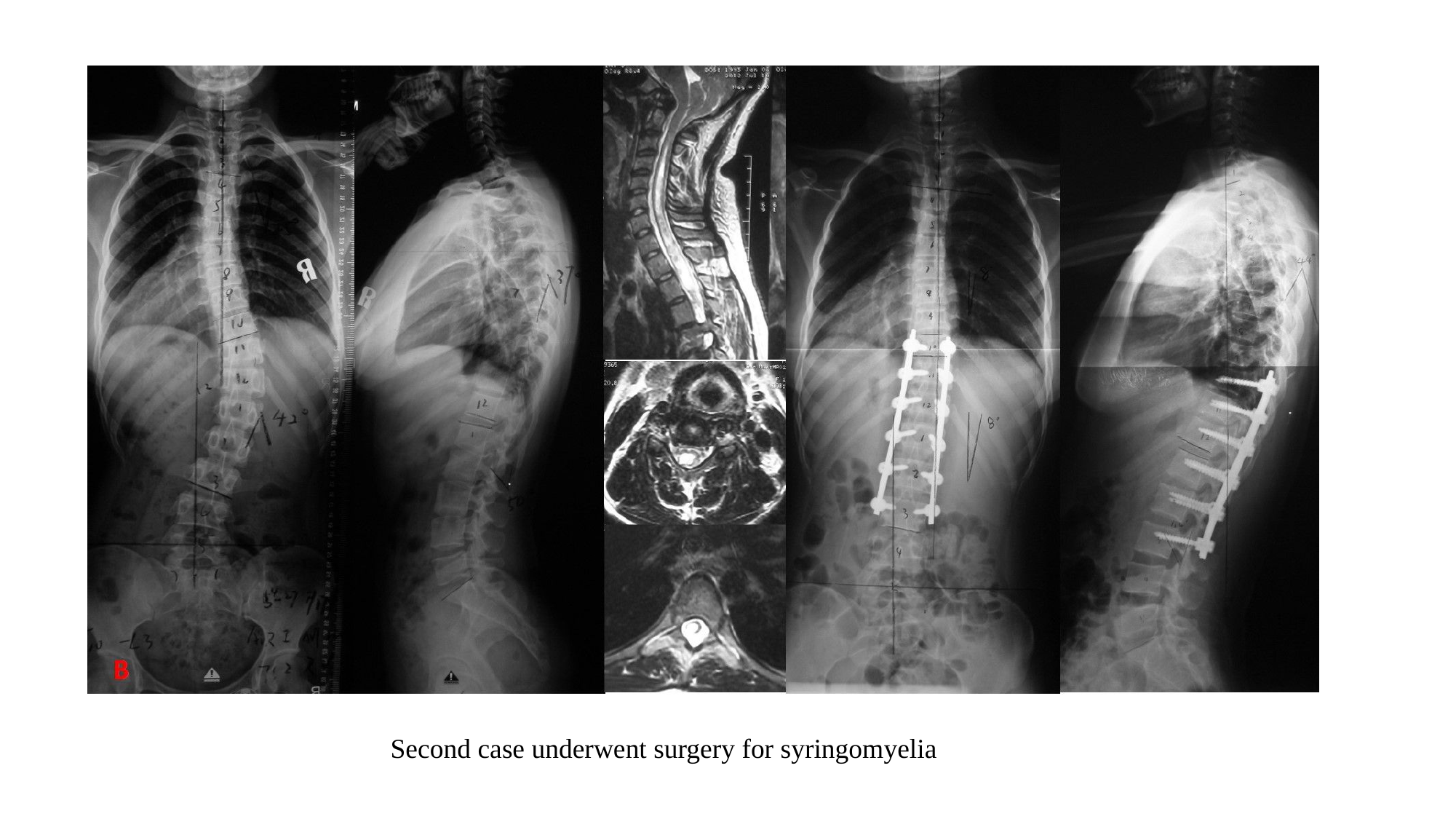

Second case underwent surgery for syringomyelia

## Slide 4
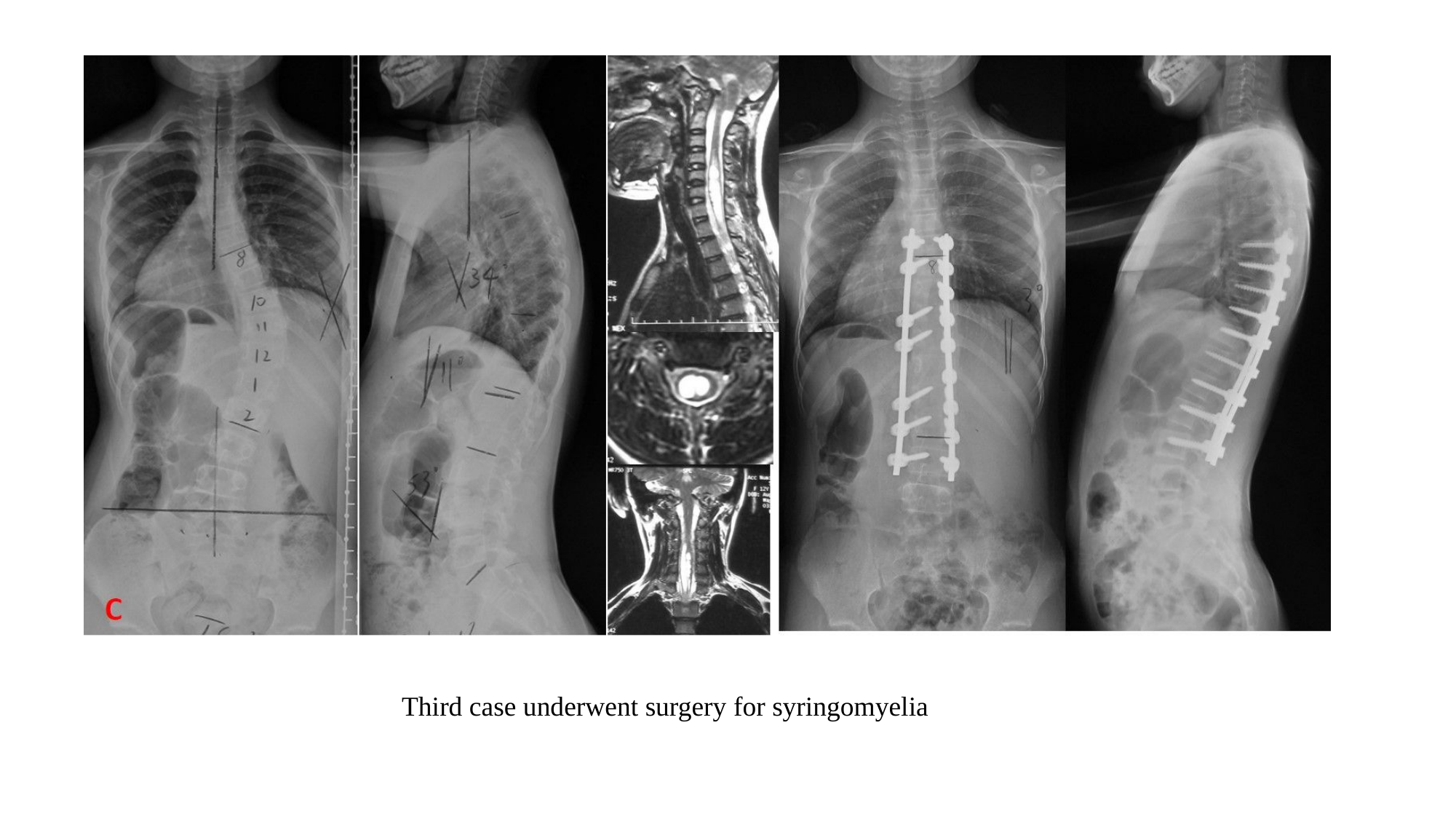

Third case underwent surgery for syringomyelia

## Slide 5
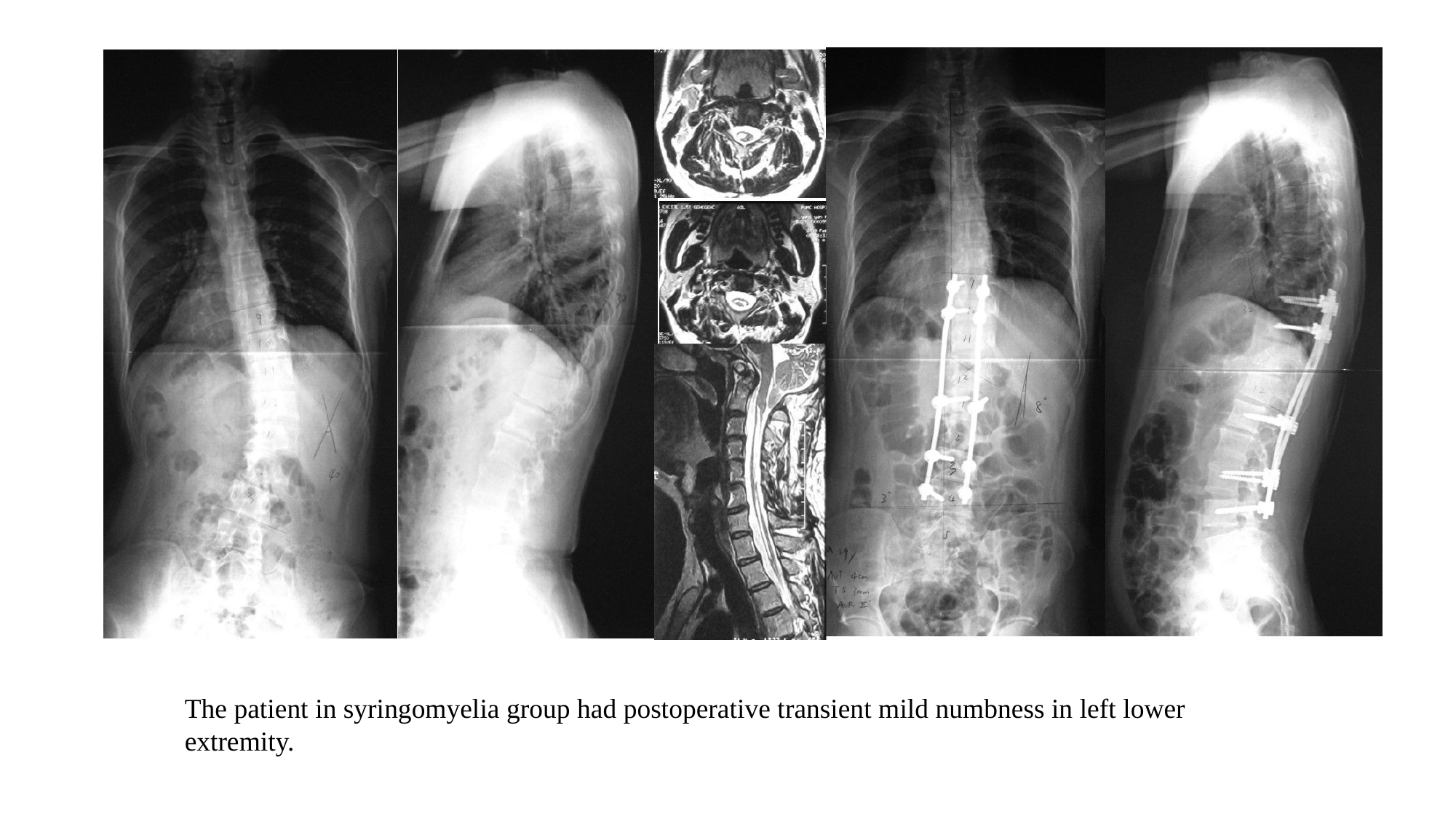

The patient in syringomyelia group had postoperative transient mild numbness in left lower extremity.
